# Tidal Breathing Pulmonary Function of Children With Allergic Rhinitis

**DOI:** 10.3389/fped.2022.808948

**Published:** 2022-03-07

**Authors:** Hui Du, Xueru Liu, Fang Peng, Hebin Chen, Yanli Wang

**Affiliations:** ^1^Department of Respiratory Medicine, Wuhan Children’s Hospital, Tongji Medical College, Huazhong University of Science and Technology, Wuhan, China; ^2^Department of Pediatrics, Children’s Digital Health and Data Center, Zhongnan Hospital of Wuhan University, Wuhan, China; ^3^Department of Pulmonary Function, Wuhan Children’s Hospital, Tongji Medical College, Huazhong University of Science and Technology, Wuhan, China

**Keywords:** allergic rhinitis (AR), tidal breathing pulmonary function, asthma, small airway function, follow-up

## Abstract

In order to investigate the characteristics of tidal breathing pulmonary function in children with allergic rhinitis, and explore its role in the relationship between allergic rhinitis and asthma, we conducted this prospective study from January 4, 2016 to January 30, 2019 in Wuhan children’s hospital. In this study, 49 children with simple allergic rhinitis were enrolled in the AR group; 50 children with allergic rhinitis concomitant with asthma were enrolled in the AR&A group; 43 healthy children were recruited in the control group. For individuals in each group, the assessment of tidal breath pulmonary function was performed after enrollment. Then participants in the AR group and control group were followed up for 1 year to observe their frequency of wheezing attacks. The parameters of tI/tE, tPTEF/tE, and VPTEF/VE of AR group were significantly higher than AR&A group (*P* < 0.001). The reduced proportion of tPTEF/tE and VPTEF/VE. in AR group were higher than that in control group (30.61% vs. 11.63%, *P* < 0.001; 24.49% vs. 11.63%, *P* < 0.001, respectively). The proportion of patients with reduced tPTEF/tE and VPTEF/VE who occurred recurrent wheezing was higher than that of patients with normal pulmonary function in AR group(*P* = 0.008). In conclusion, some children with allergic rhinitis has impaired tidal breathing pulmonary function. Tidal breathing pulmonary function test plays an important role in the diagnosis and assessment of children’s airway allergic diseases (AR and asthma).

## Introduction

Allergic diseases are one of the most common diseases in children, which often involve multiple organs such as respiratory system, skin and digestive system. In recent years, the incidence of allergic diseases has increased gradually year by year, especially in children (1–3). Among them, the main manifestations of allergic rhinitis (AR) are rhinorrhea, nasal itching, nasal congestion and sneezing, and the symptoms can last for more than 1 h every day, seriously affecting children’s quality of life (4). In some countries, the incidence of AR in children can be as high as 40%, especially in older children (5, 6). Many studies show that AR is a risk factor for the development and poor control of asthma (7), and it also impairs pulmonary function in early life.

The pulmonary function of children with single AR was impaired in varying degrees, especially the decrease of FEF 25% – FEF 75% predicted value, which affected airway hyperresponsiveness (8, 9). However, children under 5 years old could not complete the forced expiratory pulmonary function test. Tidal breathing pulmonary function test is safe, simple and suitable for children under 5 years old. This test in children with AR is of great significance in determining whether there is pulmonary function injury, whether there is recurrent wheezing and the possibility of asthma in early childhood. At present, there are few reports on whether tidal breathing pulmonary function is abnormal in young children with AR.

This study aims to explore the changes of tidal breathing pulmonary function in children aged 2–5 years old with AR, and also investigate its role in the relationship between AR and asthma. We followed up for 1 year to observe whether they are complicated with recurrent wheezing and whether they develop into asthma, so as to illustrate the relationship between changes of pulmonary function and the occurrence of asthma.

## Materials and Methods

### Subjects

**FIGURE 1:**
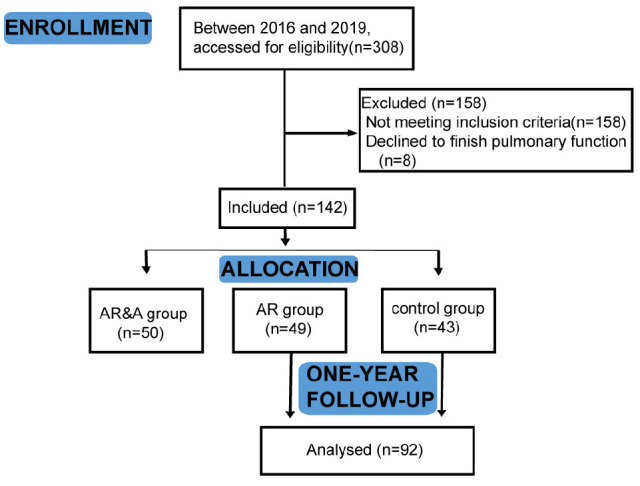
Flow chart of the study. In total, 142 participants were divided into AR group, AR&A group, and control group. After enrollment, they took the tidal breathing pulmonary function test. Participants in AR group and control group were prospectively followed up for 1 year. AR group, patients with single AR; AR&A group, AR patients combined with asthma; and control group, healthy children.

This single center prospective study was carried out from January 4, 2016 to January 30, 2019 in Wuhan children’s hospital in China. We enrolled patients with AR who presented to the outpatient clinic because of symptoms related to rhinitis. A total of 100 children hospitalized with the diagnosis of AR and 50 healthy controls aged 2–5 years old were included. Among them, patients with single AR were placed in AR group (*n* = 50), and patients combined with asthma were placed in AR&A group (*n* = 50). Besides, 50 healthy children aged 2–5 years old were recruited in control group. Eight participants did not accomplish the tidal breathing pulmonary function test and were therefore excluded from the study (Figure 1). Participants in AR group and control group were prospectively followed up for 1 year. This study was approved by the Ethics Committee of Wuhan children’s hospital (No. 2015029).

The diagnostic criteria of patients in AR group were in accordance with the “Chinese Guidelines for the Diagnosis and Treatment of allergic rhinitis in children” established in 2010 (10), and patients combined with congenital diseases of the respiratory system, tumors, immunodeficiency diseases, or cardiovascular diseases were excluded. Patients in AR&A group need to meet the criteria of “Chinese Children Bronchial asthma Diagnosis and Prevention Guidelines” in 2016 at the same time (11). Control group was recruited from children who were admitted to pediatric general health outpatient clinics.

Children who had a history of bronchopulmonary dysplasia, recurrent wheezing (≥2), chronic lung disease, congenital heart disease, previous non-respiratory infection, immunodeficiency, atopy, exposure of passive smoking, and chronic disease were excluded from control group. Individuals who suffered from acute respiratory infection or asthma exacerbation within 2 weeks before tidal breathing pulmonary function test were also excluded from the study.

### Pulmonary Function Test

Tidal breathing flow was measured with a standardized portable pediatric pulmonary function device (MasterScreen PAED; Jaeger Company, London, United Kingdom). It was carried out with one nurse and observed by pediatrician. Tidal breathing pulmonary function was measured for all participants after enrollment.

Before testing, the participant was weighed and examined, and the length was documented using a calibrated infant stadiometer. The test was performed 0.5∼2 h after eating, without obvious abdominal distension, and in a quiet sleep state (natural sleep or oral chloral hydrate 30∼40 mg/kg). The subject lies on his back with his head slightly tilted back. Cover the mouth and nose of the child with appropriate strength and press the edge of the mask tightly to avoid air leakage. During the quiet sleep period, the subjects were tested for 5 times in a row, and 20 times of tidal breathing were recorded in each time. The average value was taken to obtain the curve of flow velocity and volume and the value of each parameter.

The main parameters included expiratory time (tE), time to peak tidal expiratory flow (tPTEF), peak tidal expiratory flow (PTEF), ratio of time to reach peak tidal expiratory flow to total expiratory time (tPTEF/tE), inspiratory time (tI), volume expired before PTEF attained (VPTEF), ratio of volume until peak tidal expiratory flow to total expiratory volume (VPTEF/VE), and tidal volume (VT). All parameters were calculated by the tidal breathing analysis device computer.

### Follow-Up

Based on the tidal breathing pulmonary function, SAR group was further divided into two subgroups, the abnormal group and normal group. All the remaining participants in SAR group and control group were followed up by telephone or face-to-face interview to investigate the number of wheezing attacks in 1 year.

### Statistical Analysis

The statistical analysis was performed using the GraphPad Prism version 6.0 (GraphPad Software, San Diego, CA, United States). Student’s *T*-test was used to compare variables between two groups, while one-way analysis of variance (ANOVA) was used to compare differences among three groups for continuous variables. Categorical data were analyzed by chi-square test or Fisher’s exact test when appropriate. A *p*-value of <0.05 was considered statistically significant.

## Results

### General Characteristics

A total of 142 children completed the study. The participants’ demographic details are given in Table 1. There were no significant differences between three groups regarding age, gender, height, and weight.

**TABLE 1:** Demographic and anthropometric characteristics of the participants.

Parameter	AR (*n* = 49)	AR&A (*n* = 50)	Control (*n* = 43)	*P*-value
Gender [*n* (%)]				0.492
Male	29(36.3)	30(37.5)	21(26.3)	
Female	20(32.3)	20(32.3)	22(35.3)	
Age(month)	36.98 ± 6.39	37.54 ± 7.76	37.53 ± 6.56	0.886
Weight(kg)	15.32 ± 2.15	15.29 ± 2.27	14.95 ± 1.65	0.639
Height(cm)	93.67 ± 5.80	94.85 ± 5.63	94.44 ± 4.33	0.537

*Continuous data are shown as mean ± SD and categorical variables as number (%). AR, allergic rhinitis; AR&A, allergic rhinitis concomitant with asthma.*

### Pulmonary Function Analysis

The tidal breathing parameters of participants in three groups are summarized in Table 2. The level of tI/tE, tPTEF/tE, and VPTEF/VE were all significantly different in three groups (*P* < 0.001). The level of tI/tE, tPTEF/tE and VPTEF/VE of AR group were significantly higher than AR&A group (*P* < 0.001), and there was no statistical difference in the level of tI/tE, tPTEF/tE, and VPTEF/VE between AR group and control group (*P* = 0.377, *P* = 0.167 and *P* = 0.241, respectively). There was no difference of the VT level among three groups (*P* = 0.853).

**TABLE 2:** Comparison of tidal breathing parameters among three groups.

Parameter	AR (*n* = 49)	AR&A (*n* = 50)	Control (*n* = 43)	*P*-value
VT(ml/kg)	8.26 ± 1.03	8.39 ± 1.35	8.36 ± 0.95	0.853
tI/tE	0.76 ± 0.08	0.66 ± 0.10^a^	0.78 ± 0.07	<0.001
tPTEF/tE (%)	30.98 ± 6.84	23.35 ± 8.57^b^	32.81 ± 5.57	<0.001
VPTEF/VE (%)	32.97 ± 6.39	26.09 ± 7.51^c^	34.39 ± 4.98	<0.001

*Continuous data are shown as mean ± SD and categorical variables as number (%). AR, allergic rhinitis; AR&A, allergic rhinitis concomitant with asthma; VT, tidal volume; tI/Te, ratio of inspiratory time to expiratory time; tPTEF/tE, ratio of time to reach peak tidal expiratory flow to total expiratory time; VPTEF/VE, ratio of volume until peak tidal expiratory flow to total expiratory volume. ^a,^ ^b,^ ^c^AR group vs. AR&A group, P < 0.001.*

**FIGURE 2:**
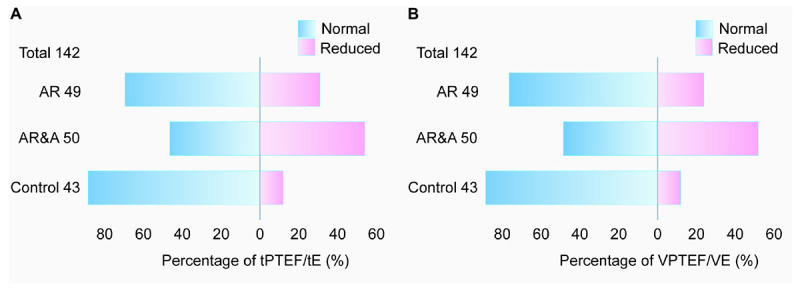
Comparison of abnormal proportion of **(A)** tPTEF/tE and **(B)** VPTEF/VE among three groups. The total number of participants is 142, including 49 in AR group, 50 in AR&A group, and 43 in control group. The proportions of abnormal tPTEF/tE and VPTEF/VE were different among the three groups, the highest in AR&A group, the second in AR group and the lowest in control group. Blue, percentage of participants with normal tPTEF/tE or VPTEF/VE; Pink, percentage of participants with reduced tPTEF/tE or VPTEF/VE. AR, allergic rhinitis; AR&A, allergic rhinitis concomitant with asthma; tPTEF/tE, ratio of time to reach peak tidal expiratory flow to total expiratory time; VPTEF/VE, ratio of volume until peak tidal expiratory flow to total expiratory volume.

As shown in Figure 2, the proportion of abnormal tPTEF/tE and VPTEF/VE in each group was analyzed. In AR group, there were 15 cases with reduced tPTEF/tE and 12 cases with reduced VPTEF/VE. There were 27 cases with reduced tPTEF/tE and, 26 cases with reduced VPTEF/VE in AR&A group, while there were 5 cases with reduced tPTEF/tE and VPEF/VE in control group. The reduced proportion of tPTEF/tE and VPTEF/VE in AR group were significantly higher than that in control group (30.61% vs. 11.63%, *P* < 0.001; 24.49% vs. 11.63%, *P* < 0.001, respectively).

### Association Between Pulmonary Function of Allergic Rhinitis Patients and the Frequency of Wheezing Attacks

**FIGURE 3:**
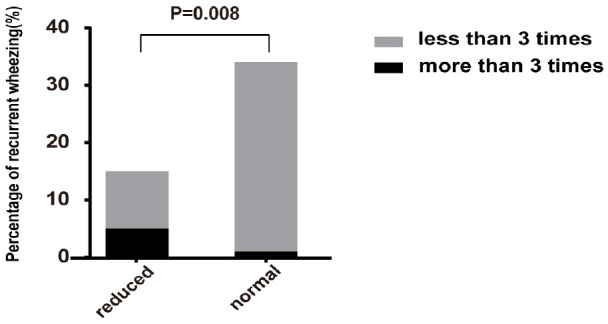
Comparison of proportion of recurrent wheezing in reduced group and normal group of AR patients. The percentage of recurrent wheezing in reduced group was significantly higher than that in normal group (*P* = 0.008). Reduced, patients with reduced pulmonary function in AR group; normal, patients with normal pulmonary function in AR group.

The AR group was further divided into the reduced group and the normal group according to whether the pulmonary function was normal. 15 cases in the reduced group, 34 cases in the normal group, and 43 cases in control group were followed up for 1 year. In reduced group, 5 cases (33.33%) had recurrent wheezing attacks in the following year (more than 3 times), and 10 cases had no wheezing attack or wheezing less than three times. While only 1 case (2.94%) in the normal group had recurrent wheezing attacks in the following year, and the other 33 cases had no wheezing attacks. As shown in Figure 3, there was significant difference in the frequency of wheezing attacks between the two subgroups (*P* = 0.008). There was no case in control group who recurrently wheezing during the 1-year follow-up period.

## Discussion

The incidence of AR in children is high, especially in young children. AR and asthma are common diseases that frequently occur together. This concept has been referred to in the literature as united airway disease (12). Epidemiological studies have shown that the majority of patients with asthma have concomitant rhinitis and the presence of rhinitis is an increased risk factor for development of asthma (13). However, how we recognize patients with AR who may progress to asthma early is still not clear. Pulmonary function test is safe, simple and accurate, which plays an important role in the diagnosis and assessment of asthma. Previous studies found that reduced pulmonary function occurred earlier than asthma in infancy, and it can predict asthma or persistent abnormal pulmonary function in adulthood (14). In order to recognize patients with AR who are likely to develop to asthma with pulmonary function test in early period, we carried out this study.

Pulmonary function test can distinguish the lesion site. AR belongs to the upper respiratory tract disease concerned the lesion site. In theory, there should not be small airway dysfunction without asthma or lower respiratory tract infection. However, some studies found that some simple AR patients have small airway obstruction and/or high airway reactivity through forced expiratory pulmonary function test (8). The incidence of asthma in AR patients with small airway dysfunction is higher, and it’s more difficult to control this kind of asthma (9, 15–17). This conclusion is consistent with the above corollary. So, studying the changes of pulmonary function in children with AR is helpful for early detection of small airway obstruction and airway hyperresponsiveness, and it may predict the occurrence and development of asthma, which is of great significance for the prevention and treatment of AR and asthma.

Due to age limitation, children under 5 years old usually cannot complete the forced expiratory pulmonary function test, tidal breathing pulmonary function test or pulse concussion pulmonary function test can be used in clinical practice instead. At present, it was not very clear that whether the tidal breathing pulmonary function is impaired in young children with AR. Therefore, this study included 99 children aged 2–5 years old with AR. We found that the tI/tE, tPTEF/tE, and VPTEF/VE of patients in AR&A group were lower than those of the other two groups. Although there was no significant difference in tPTEF/tE and VPTEF/VE between AR group and control group, the proportion of reduced tPTEF/tE and VPTEF/VE in AR group was significantly higher than that in control group. tPTEF/tE and VPTEF/VE are well correlated with obstruction of small airways, mainly involving bronchioles less than 2 mm in diameter, and the lower the parameters are, the more severe the degree of small airway obstruction is (18). This study showed that lower respiratory tract obstruction was more obvious in AR&A group. Because asthma itself was mainly involved in small airway. The reduced tPTEF/tE and VPTEF/VE in AR group, suggesting that patients with simple AR may also have small airway dysfunction without lower respiratory tract infection or asthma. The main reason may be that AR and asthma share the same pathogenesis and pathophysiological changes.

Furthermore, we followed up the participants of AR group and the control group for 1 year. We found that the proportion of participants with AR who occurred recurrent wheezing was higher than that of the control group, and it’s also higher in patients with reduced tPTEF/tE and VPTEF/VE than that of patients with normal pulmonary function in AR group. It suggested that the impaired pulmonary function in patients with AR might increase the possibility of concomitant with asthma. Previous studies showed that AR is a risk factor for asthma, and the incidence of asthma is significantly higher than that of the normal population without intervention (19, 20). However, due to the limitation of follow-up time in this study, further studies are needed to confirms the correlation between AR and asthma. Clinicians should also be alert to AR patients with reduced tPTEF/tE and VPTEF/VE.

There are several relevant limitations to this study. Our findings are based on single-center study. There may be varying degrees of bias due to small sample size. But our results make it possible to monitor pulmonary function in young children with AR. And the follow-up is not long enough. Further we will carry out a multicenter study and extend the follow-up time to fully understand the changes in pulmonary function during the course of AR in children.

In conclusion, the tidal breathing pulmonary function test plays an important role in the diagnosis and assessment of children’s airway allergic diseases (AR and asthma). It can detect airway dysfunction in children with AR in early period, so as to predict the severity and prognosis of asthma, which can provide guidance for clinicians.

## Data Availability Statement

The original contributions presented in the study are included in the article/supplementary material, further inquiries can be directed to the corresponding author.

## Ethics Statement

The studies involving human participants were reviewed and approved by the Ethics Committee of Wuhan Children’s Hospital (No. 2015029). Written informed consent to participate in this study was provided by the participants’ legal guardian/next of kin. Written informed consent was obtained from the individual(s), and minor(s)’ legal guardian/next of kin, for the publication of any potentially identifiable images or data included in this article.

## Author Contributions

YW conceived the study. HD, XL, FP, and HC collected the data. HD and YW analyzed the data. HD drafted the manuscript. All authors participated in the study design, gave comments on the earlier versions of the manuscript, and edited the manuscript and approved the final version.

## Conflict of Interest

The authors declare that the research was conducted in the absence of any commercial or financial relationships that could be construed as a potential conflict of interest.

## Publisher’s Note

All claims expressed in this article are solely those of the authors and do not necessarily represent those of their affiliated organizations, or those of the publisher, the editors and the reviewers. Any product that may be evaluated in this article, or claim that may be made by its manufacturer, is not guaranteed or endorsed by the publisher.
